# A 13-Gene Signature Based on Estrogen Response Pathway for Predicting Survival and Immune Responses of Patients With UCEC

**DOI:** 10.3389/fmolb.2022.833910

**Published:** 2022-04-26

**Authors:** Yimin Li, Ruotong Tian, Jiaxin Liu, Chunlin Ou, Qihui Wu, Xiaodan Fu

**Affiliations:** ^1^ Department of Pathology, Fudan University Shanghai Cancer Center, Shanghai, China; ^2^ Department of Oncology, Shanghai Medical College, Fudan University, Shanghai, China; ^3^ Department of Pharmacology, School of Basic Medical Sciences, Shanghai Medical College, Fudan University, Shanghai, China; ^4^ Department of Pathology, School of Basic Medical Sciences, Central South University, Changsha, China; ^5^ Department of Pathology, Xiangya Hospital, Central South University, Changsha, China; ^6^ National Clinical Research Center for Geriatric Disorders, Xiangya Hospital, Changsha, China; ^7^ Department of Obstetrics and Gynecology, Xiangya Hospital, Central South University, Changsha, China

**Keywords:** UCEC, TCGA, estrogens, immune infiltration, prognosis

## Abstract

**Background:** Accumulating evidence suggests that anti-estrogens have been effective against multiple gynecological diseases, especially advanced uterine corpus endometrial carcinoma (UCEC), highlighting the contribution of the estrogen response pathway in UCEC progression. This study aims to identify a reliable prognostic signature for potentially aiding in the comprehensive management of UCEC.

**Methods:** Firstly, univariate Cox and LASSO regression were performed to identify a satisfying UCEC prognostic model quantifying patients’ risk, constructed from estrogen-response-related genes and verified to be effective by Kaplan-Meier curves, ROC curves, univariate and multivariate Cox regression. Additionally, a nomogram was constructed integrating the prognostic model and other clinicopathological parameters. Next, UCEC patients from the TCGA dataset were divided into low- and high-risk groups according to the median risk score. To elucidate differences in biological characteristics between the two risk groups, pathway enrichment, immune landscape, genomic alterations, and therapeutic responses were evaluated to satisfy this objective. As for treatment, effective responses to anti-PD-1 therapy in the low-risk patients and sensitivity to six chemotherapy drugs in the high-risk patients were demonstrated.

**Results:** The low-risk group with a relatively favorable prognosis was marked by increased immune cell infiltration, higher expression levels of HLA members and immune checkpoint biomarkers, higher tumor mutation burden, and lower copy number alterations. This UCEC prognostic signature, composed of 13 estrogen-response-related genes, has been identified and verified as effective.

**Conclusion:** Our study provides molecular signatures for further functional and therapeutic investigations of estrogen-response-related genes in UCEC and represents a potential systemic approach to characterize key factors in UCEC pathogenesis and therapeutic responses.

## Introduction

Uterine corpus cancer is the sixth most commonly diagnosed cancer in women globally, with an estimated 417,000 new cases and 97,000 deaths in 2020 worldwide (Sung et al., 2021). Among them, uterine corpus endometrial carcinoma (UCEC), which originates from the uterine epithelium, is the most common uterine cancer type (Urick and Bell, 2019). In 1983, considering the role of endocrine and metabolic disturbances in the pathogenesis of endometrial carcinoma, Bokhman JV (Bokhman, 1983) postulated two different types: estrogen-dependent Type I and estrogen-independent Type II. The prognosis for Type I is favorable, with a greater than 85% 5-years disease-free survival rate. In contrast, Type II is associated with a poor response to hormonal therapy and a relatively poor outcome (Brinton et al., 2013). However, this classification method has severe limitations in routine clinical practice due to high subjectivity and low reproducibility, and it fails to evaluate prognosis and guide individualized treatment well (Huvila et al., 2021). With the aim of more precise classification, based on multi-omics characterization, The Cancer Genome Atlas (TCGA) took tumor mutation burden into account. It classified endometrial carcinomas into four molecular subtypes with prognostic significance, including POLE ultra-mutated, microsatellite instability hypermutated (MSI-H), copy-number low (CN-low), and copy-number high (CN-high) (Cancer Genome Atlas Research et al., 2013). Theoretically, TCGA classification can assist in clinical determination. Whereas poor maneuverability of the method leads to limited clinical application. Therefore, it is necessary to explore tumor molecular profiling to overcome difficulties associated with incorporating molecular subtyping into the clinic and improve our capacity to predict patients’ prognoses.

Estrogens are a group of steroid compounds that function in a myriad of physiologic and pathologic processes (Markov et al., 2017). Estrogens exert both genomic and non-genomic biological effects, mediated traditionally by two cognate estrogen receptors (ERs): estrogen receptor α (ERα) or estrogen receptor β (ERβ), which belong to the nuclear receptor superfamily (Mosselman et al., 1996; Chen et al., 2008). Estrogen and its receptors orchestrate the development of malignant tumors such as breast and gynecologic cancers, endocrine gland cancers, digestive cancers, and lung carcinoma (Chen et al., 2008; Liang and Shang, 2013; Rothenberger et al., 2018). It is worth noting that the continued annual increase in incidence and disease-related mortality of endometrial carcinomas (Lortet-Tieulent et al., 2018). The 5-years survival rate of stage IV endometrial carcinoma can only reach 20% (Braun et al., 2016). Meanwhile, the treatment options for endometrial carcinoma have been limited and relatively unchanged in the past several years (Ito et al., 2007; Rodriguez et al., 2019). Despite a high proportion of endometrioid ECs being ER and/or PR positive, endocrine therapy is only effective in a minority of women with EC, and ultimately patients progress with resistance to treatment. A greater understanding of ER and PR biology may help identify patient populations who will derive benefits and strategies for new therapeutic options. Obtaining a more accurate understanding of how estrogen functions in EC might provide significant insights into the development of therapies that block estrogen pathways.

Immunotherapy has become a potential therapeutic strategy for treating advanced cancer in recent years (Rodriguez et al., 2019). Currently, immune checkpoint inhibitors (ICIs) targeting Cytotoxic T-lymphocyte antigen 4 (CTLA-4) and Programmed cell death 1 receptor (PD1, PDCD1) are undergoing clinical evaluation (Blackburn et al., 2009; Rothenberger et al., 2018; Forschner et al., 2019). In a subset of patients with heavily pretreated advanced PD-L1-positive EC, pembrolizumab, a humanized monoclonal antibody that targets PD-1, demonstrated a favorable safety profile and durable antitumor activity (Ott et al., 2017). Unarguably, ICIs significantly improve the treatment of advanced cancers and benefit EC patients’ survival rates compared to conventional chemotherapy. Nevertheless, only around 20–35% of typical response rates to these therapies cannot be overlooked (Patel and Kurzrock, 2015; Reck et al., 2016; Wolchok et al., 2017; Rothenberger et al., 2018). Estrogen receptors are widespread in many kinds of cells involved in innate and adaptive immune responses and the formation of the tumor microenvironment. Recent research implicates estrogen as a potential mediator of immunosuppression by modulating protumor responses independent of direct activity on tumor cells (Rothenberger et al., 2018). Susanne Svensson et al. showed that estradiol enhanced macrophage influx and angiogenesis by releasing CCL2, CCL5, and vascular endothelial growth factor (Svensson et al., 2015). Also, several animal and human studies revealed that elevated estrogen enhanced Th2 responses and thus exhibited cancer-promoting activity (Khan and Ansar Ahmed, 2015). Furthermore, estrogen enhances immunosuppression by inhibiting NK and CTL-mediated tumor cell elimination (Jiang et al., 2006; Jiang et al., 2007). In spite of this, it remains unclear whether estrogen response-related genes influence the EC microenvironment and clinical outcomes.

This study captured the expression profiles of 200 genes related to estrogen-response from the TCGA-UCEC dataset. The single sample Gene Set Enrichment Analysis (ssGSEA) algorithm was performed to obtain the enrichment scores of the estrogen response pathway of each UCEC patient. Subsequently, correlation analysis among estrogen response ssGSEA score, prognosis, and clinical features of patients was performed. Next, an estrogen-response-related risk model was established, and the UCEC patients were classified into low- and high-risk groups in light of their risk scores. Additionally, we established a nomogram that integrated the prognosis model with clinicopathological factors to predict the overall survival of patients with UCEC. Finally, integrated analyses of pathway enrichment, immune landscape, somatic mutation and copy number variation (CNV), and immuno-/chemotherapeutic response prediction were conducted in the two risk groups.

## Materials and Methods

### Data Acquisition and Preprocessing

The transcriptome data (FPKM values) of the TCGA-UCEC was downloaded from the UCSC Xena browser (https://xena.ucsc.edu/public-hubs). The mRNAs with a normalized count equal to or above 1 in at least 10% of the samples were left for further analysis (Lu et al., 2019). The corresponding UCEC clinical parameters were downloaded from the UCSC Xena browser and cBioPortal (http://www.cbioportal.org/datasets). 570 samples, including 535 tumor samples and 35 normal samples, were obtained with integrated clinical information. These tumor samples (the Entire set) were randomly classified into the Training set (*n* = 268) and the Validation set (*n* = 267). The Training set was used for constructing the prognostic model, while both the Entire set and the Validation set were used for validation. The data of somatic mutations and CNV was obtained through the “TCGAbiolinks” package. The mutation data was processed and visualized using the “maftools” package. The CNV data was processed and visualized using GISTIC 2.0.

### Identification of Hallmarks With Prognostic Significance

Gene sets of cancer-related hallmarks were downloaded from the Molecular Signatures Database (MSigDB) (https://www.gsea-msigdb.org/gsea/msigdb/index.jsp). To obtain the enrichment scores of gene sets with these hallmarks, we used the ssGSEA algorithm according to our previous studies (Tian et al., 2021). Further, univariate regression analysis was performed to explore the correlation between hallmarks and patients’ overall survival (OS) or disease-free survival (DFS).

### Construction of Prognostic Signature With LASSO Regression Model

A Univariate Cox regression analysis was performed to evaluate the correlation between OS and differentially expressed genes in the estrogen response pathway. According to the criteria of *p* -value < 0.05, 34 genes significantly associated with OS were selected out for the least absolute shrinkage and selection operator (LASSO) Cox regression by using the “glmnet” package with the penalty parameter estimated by 10-fold cross-validation. So far, the UCEC prognostic signature consisting of 13 genes has been established. The risk score calculation formula is:

$$Risk\;score=\overset{n}{\underset{i=1}{\sum }}\text{Coefi∗}x_{i}$$

(Coefi means the coefficients, $x_{i}$ is the FPKM value of each prognostic-related gene).

According to the median risk score, UCEC patients from the Training, Validation, and Entire set were divided into low- and high-risk groups. A time-dependent ROC and Kaplan–Meier curve analysis were performed to test the validity of the prognostic signature. Further Univariate and multivariate Cox regression analyses were performed to validate the independent role of the prognostic signature.

### Functional and Pathway Enrichment Analyses

We compared the differences in biological characteristics between the low- and high-risk groups in the TCGA-UCEC dataset with *p -value* < 0.05 and *t -value* > 2 as thresholds (PMID: (Hanzelmann et al., 2013). Gene ontology (GO) and the Kyoto Encyclopedia of Genes and Genomes (KEGG) pathway enrichment analyses were performed using the “clusterProfiler” package (Yu et al., 2012).

### Comparison of Immune Status in the Low- and High-Risk Groups

These ssGSEA scores of 24 immune cell types were obtained using the “Gene Set Variation Analysis (GSVA)” package with default parameters. Differences in these scores were analyzed in the low- and high-risk groups. Specifically, the ESTIMATE algorithm was used to evaluate Tumor Purity, ESTIMATE Score, Immune Score, and Stromal Score. The differential expression levels of human leukocyte antigen (HLA) family members and immune checkpoint biomarkers between the high- and the low-risk group were further analyzed.

### Prediction of Patients’ Responses to Immunotherapy and Chemotherapy

SubMap was used to compare the similarity of expression profiles, and this feature can be reflected as a treatment response. To predict the sensitivity of each subgroup to immunotherapy, we used a subclass mapping algorithm to compare the similarity of the gene expression profiles of the subgroups to those of melanoma patients receiving checkpoint blockade against programmed cell death protein-1 (PD1) or cytotoxic T lymphocyte antigen-4 (CTLA4) (Roh et al., 2017; Tang et al., 2020). Based on the Genomics of Drug Sensitivity in Cancer (GDSC) (https://www.cancerrxgene.org/), UCEC patients’ sensitivity to six chemotherapeutic agents was estimated, including docetaxel, lenalidomide, doxorubicin, cisplatin, vinorelbine, and gefitinib, by using the “pRRophetic” package (Lu et al., 2019), which is a popular enrichment algorithm extensively utilized in medical studies (Liu et al., 2021; Liu et al., 2022a; Liu et al., 2022b; Liu et al., 2022c).

### Statistical Analysis

Statistical analyses were implemented using R software (version 3.6.3). Survival analysis was performed by the Kaplan-Meier method using the “survminer” package, with the log-rank test used for comparisons. The prognostic nomogram was built based on the result of the multivariate Cox proportional hazards regression analysis, which was used to predict the 1-, 3- and 5-years OS by representing the sum of points for each factor. Next, the calibration curves were used to assess the relationship between the predicted probabilities and actual outcomes, and the calibration was evaluated by bootstrapping 1,000 times. Further, the time-dependent area under the ROC curve (AUC) were calculated to evaluate the accuracy values of prognostic models. Also, decision curve analysis (DCA) was used to evaluate the clinical benefit of the nomogram model by quantifying the net benefit of nomogram-assisted decisions. The “limma” package was used to identify differentially expressed genes with | log2(Fold change (FC)) | > 0.5 and adj *p* -value < 0.05 as the cut-off. The Chi-square test, or Fisher’s exact test, was used for categorical data. Unpaired Student’s *t*-test, Wilcoxon rank-sum test, or Kruskal–Wallis tests was used for continuous data. All *p* -values of statistical data were based on two-sided statistical tests, and data with *p* < 0.05 was considered to be statistically significant.

## Results

### Estrogen Response Pathway ssGSEA Score Was Correlated With Clinical Features of Patients With UCEC

**FIGURE 1:**
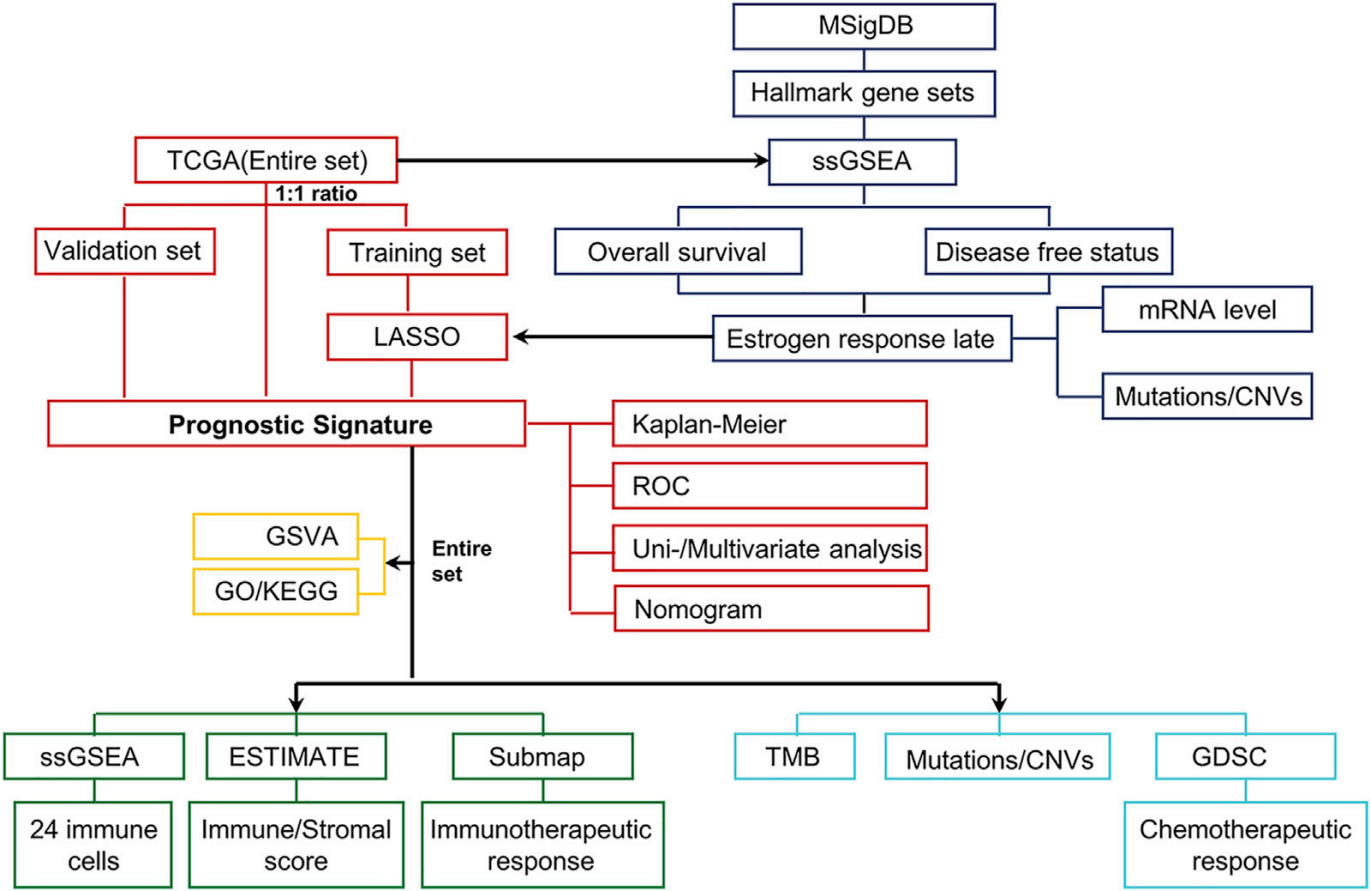
Schematic workflow for establishing the estrogen-response-related risk model and a multidimensional evaluation of patients in different risk groups. First, the transcriptome data and the corresponding clinical characteristics data of UCEC were downloaded from the UCSC Xena browser and cBioPortal. These tumor samples (the Entire set) were then randomly classified into the Training set (*n* = 268) and the Validation set (*n* = 267). The ssGSEA scores of various hallmarks were calculated based on the transcriptome profiling of the Entire set and gene sets of MSigDB using ssGSEA. We identified that the “Estrogen response late” ssGSEA score was the most significant protective factor for OS and DFS in UCEC. Subsequently, the LASSO Cox analysis model was used to identify and construct a prognostic gene signature in the Training set, further verified in the Validation set and Entire set. Next, UCEC patients from the TCGA dataset were divided into low- and high-risk groups according to the median risk score. The Kaplan–Meier curves, ROC curves, univariate and multivariate Cox regression, and nomogram were performed to identify the independent predictors of OS. To elucidate differences in biological characteristics between the two risk groups, pathway enrichment, immune landscape, genomic alterations, and therapeutic responses were evaluated to satisfy this objective.

**FIGURE 2:**
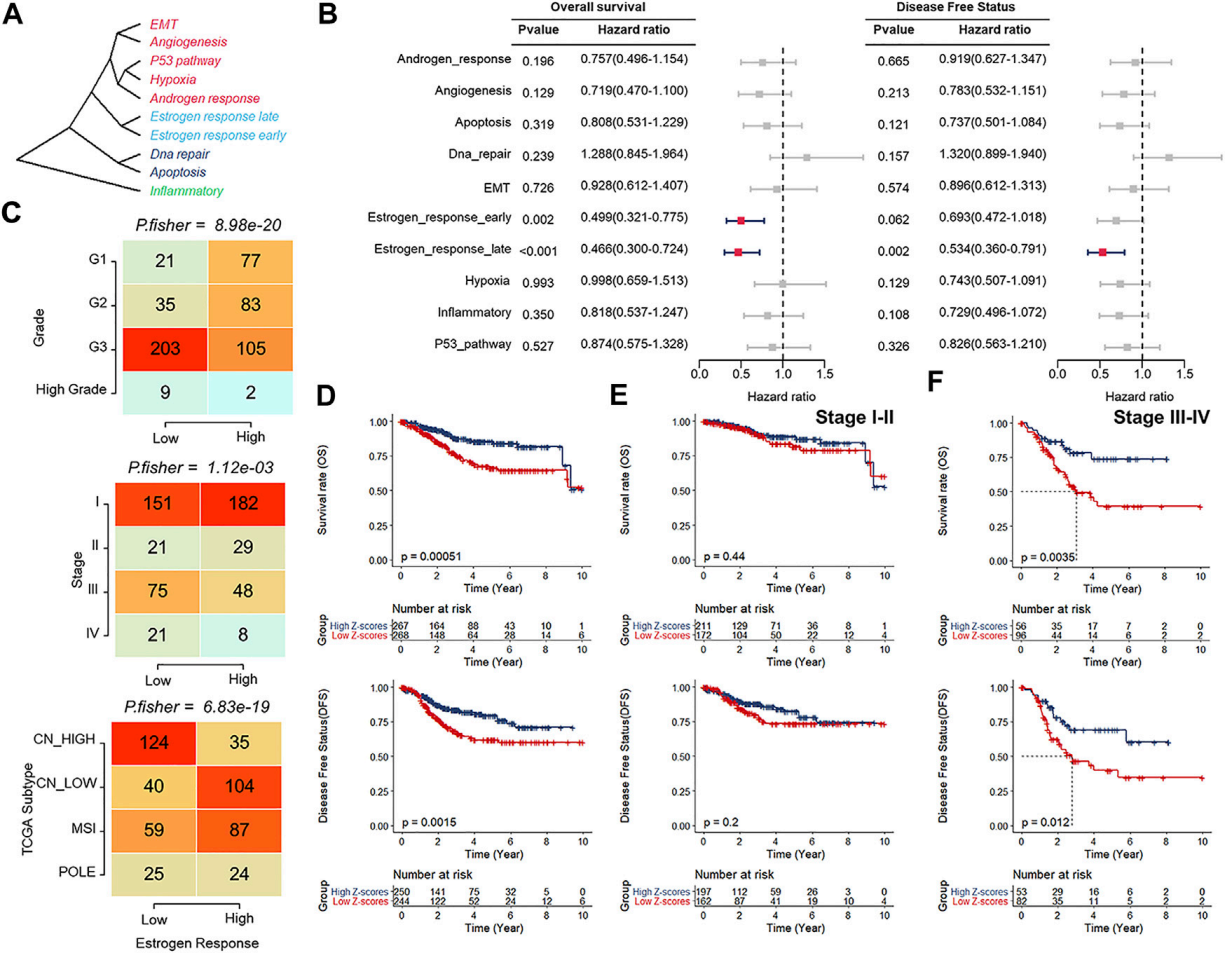
Estrogen response pathway ssGSEA score was correlated with clinical features of patients with UCEC. **(A)** An unrooted clustering dendrogram depicting the distance between different hallmarks of cancer. **(B)** Univariate Cox regression analysis indicating cancer-related hallmarks that might affect UCEC progression. **(C)** Contingency tables demonstrating the correlation between the “Estrogen response late” ssGSEA score and pathological parameters, including tumor grade (upper), clinical stage (middle), and TCGA molecular subtypes (bottom). **(D–F)** Kaplan–Meier survival analyses of the low and high ssGSEA score groups patients at all stages **(D)**, stage I ∼ II **(E)**, and stage III ∼ IV **(F)**.

The overview workflow of our research is shown in Figure 1. The gene sets of cancer-related hallmarks were downloaded from MSigDB, and the activation levels of these hallmarks in each sample from TCGA-UCEC were quantified using the ssGSEA method based on transcriptome profiling data. An unrooted clustering dendrogram was generated through hierarchical clustering analysis to show the distance between these hallmarks (Figure 2A). We performed a univariate Cox regression analysis to determine which hallmarks might affect UCEC progression, demonstrating that the “Estrogen response late” ssGSEA score was the most significant protective factor for OS and DFS in UCEC (Figure 2B). Further, we divided UCEC patients into two groups (the low and the high ssGSEA score groups) by the median ssGSEA score of “Estrogen response late”. Contingency tables demonstrated the correlation between the “Estrogen response late” ssGSEA score and pathological parameters (stage, grade, and TCGA molecular subtype) in TCGA-UCEC (Figure 2C). Kaplan–Meier survival analysis revealed that patients in the high ssGSEA score group had notably more favorable OS and DFS than those in the low ssGSEA score group (Figure 2D). Furthermore, we evaluated this classification based on the estrogen response pathway at different UCEC stages regarding patient outcomes. The results did reveal significant differences in OS as well as DFS between the two groups at stage III ∼ IV, whereas the difference in the prognosis of patients at stage I ∼ II was significant but not obvious (Figures 2E,F). It meant that this classification might be more discriminative in assessing the outcomes of patients at stage III ∼ IV. The above data indicated that the estrogen response pathway ssGSEA score could be a significant predictive factor for OS and DFS of UCEC patients, specifically in their senior stages.

### Analyses of the Differentially Expressed Genes and Genomic Alterations in the Estrogen Response Pathway

**FIGURE 3:**
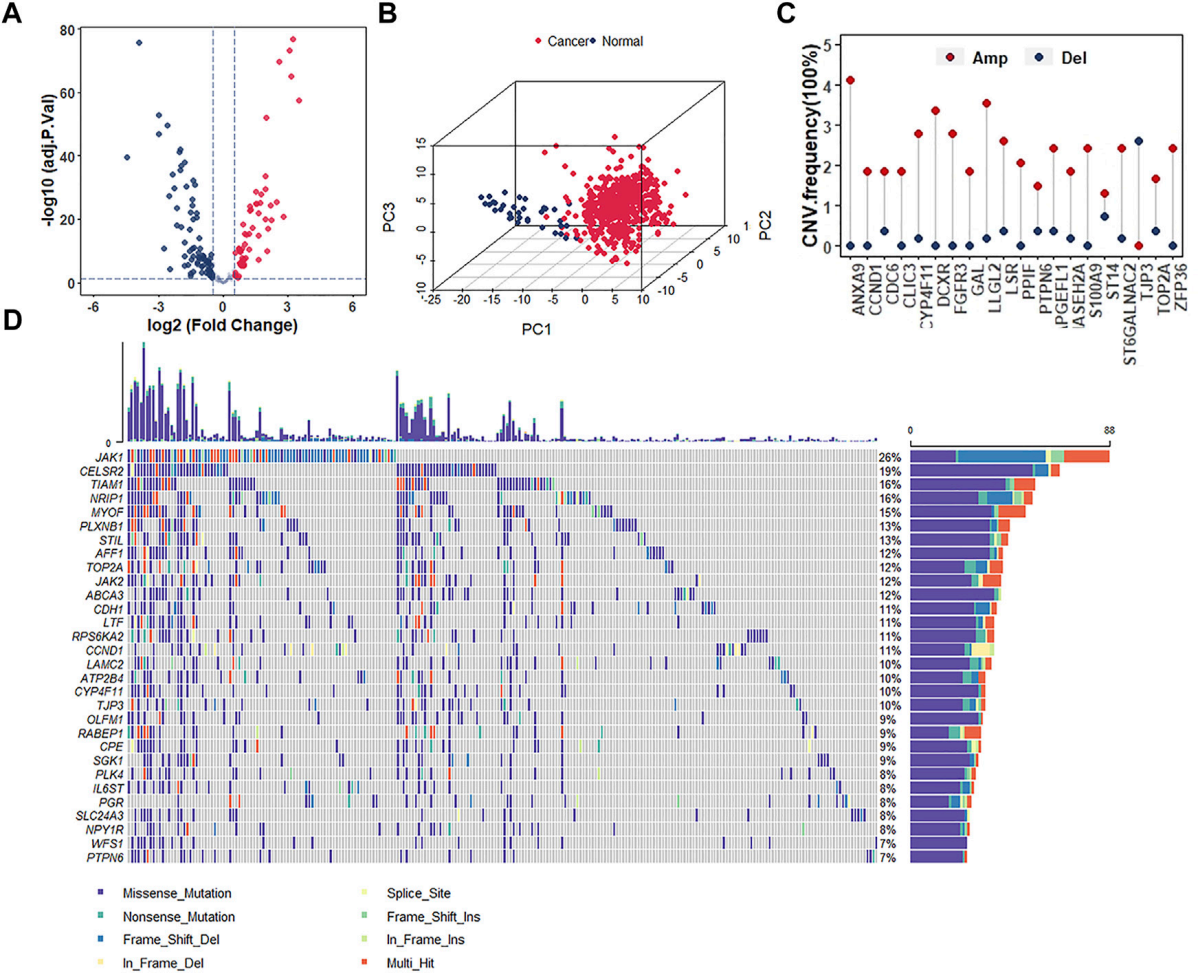
Analyses of the differentially expressed genes and genomic alterations in the estrogen response pathway. **(A)** Volcano plot representing the DEERGs in estrogen response pathway between normal and UCEC samples. **(B)** Principal component analysis (PCA) indicating the expression profiles of 132 DEERGs s being able to distinguish UCEC tumors from normal samples. **(C)** The CNV frequency of the top 20 DEERGs in the estrogen response pathway in TCGA-UCEC. **(D)** The mutation frequency of the top 30 DEERGs in the estrogen response pathway in TCGA-UCEC.

Since the protective role of estrogen response pathway ssGSEA score has been identified, it deserves further exploration of the role of key genes in the estrogen response pathway in UCEC development as well as prognosis and their alterations within the genomes. We analyzed a total of 200 estrogen-response-related genes (ERGs) to investigate whether estrogen response has a bearing on the prognosis of UCEC patients. According to the criteria of |log2FC| > 0.5 and adj *p* -value < 0.05, the expression of these 200 genes was analyzed between UCEC and normal samples. We found 132 differentially expressed ERGs (DEERGs), of which 57 and 75 were up-regulated and down-regulated, respectively (Figure 3A). Based on the expression of these DEERGs, we could completely distinguish UCEC samples from normal samples (Figure 3B). Next, we analyzed the occurrence of CNV and somatic mutations of these genes in UCEC samples. The top 20 frequent CNV and the top 30 most frequently mutated genes were illustrated (Figures 3C,D). Most DEERGs were discovered with altofrequent CNV amplifications, while only TJP3 had a widespread CNV deletion (Figure 3C). Meanwhile, ANXA9 exhibited the highest mutation frequency, followed by LLGL2 (Figure 3D). These data indicated that abnormal expression and mutation of these DEERGs contribute to the oncogenesis and progression of UCEC.

### Establishment of the UCEC Prognostic Signature Consisting of 13 Genes in the Estrogen Response Pathway

**FIGURE 4:**
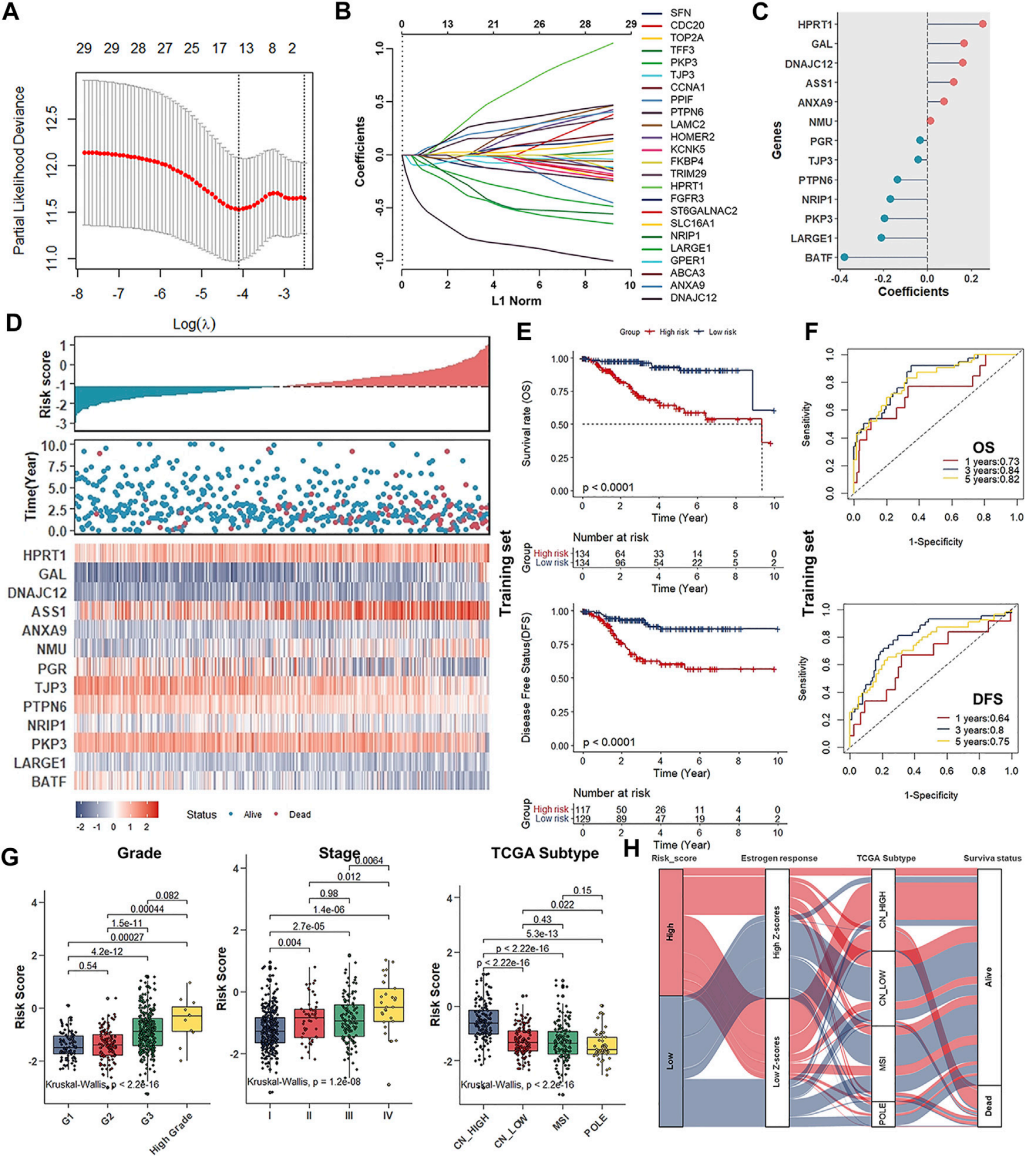
Establishment of the UCEC prognostic signature consisting of 13 genes in the estrogen response pathway. **(A)** LASSO regression identifying robust prognostic genes. **(B)** Distribution of LASSO coefficients for 34 genes in the 10-fold cross-validation. **(C)** Forest plot of the prognostic ability of the 13 DEERGs included in the prognostic signature. **(D)**. The risk score plots, OS status plots, and heatmaps of these 13 genes in the TCGA-UCEC. **(E)** Kaplan–Meier survival analysis of the low- and high-risk groups patients in the Training set. **(F)**. ROC curves analysis according to the 1, 3, 5-years survival of the area under the AUC value in the Training set. **(G)** Differences in risk scores of patient groups with different tumor grades (left), clinical stage (middle), and TCGA molecular subtypes (right). **(H)** Alluvial diagram establishing associations among risk groups, estrogen response ssGSEA score groups, TCGA molecular subtypes, and survival outcomes.

Given that only the UCEC-TCGA public dataset covers detailed clinical parameters and prognostic information, we entirely randomly grouped patients from UCEC-TCGA (n = 535) into two sets: the Training set (n = 268) and the Validation set (n = 267). To screen out DEERGs with potential prognostic value and develop one robust model to predict the prognosis of patients with UCEC, we performed a univariate Cox regression analysis in the entire set and identified 34 genes significantly associated with OS according to the criteria of *p* -value < 0.05 (Table 1). Next, we adopted the least absolute shrinkage and selection operator (LASSO) regression algorithm in the Training set for narrowing down and identifying critical candidates for further study (Figures 4A,B). At this point, we had established the UCEC prognostic signature consisting of 13 DEERGs, and the risk score was calculated as the following formula:

Risk score=−-0.196∗Exp PKP3+0.014∗Exp NMU-0.040∗Exp TJP3-0.135∗ExpPTPN6-0.379∗Exp BATF+0.168∗Exp GAL+0.255∗Exp HPRT1-0.170∗Exp NRIP1+0.120∗Exp ASS1-0.209∗Exp LARGE1+0.077∗Exp ANXA9+0.162∗Exp DNAJC12-0.032∗Exp PGR

**TABLE 1:** 34 genes significantly associated with OS by univariate Cox proportional hazards regression.

Gene	HR	HR.95L	HR.95H	*p* Value
PGR	0.728359367	0.639924061	0.829016129	1.59E-06
ASS1	1.341884582	1.180678725	1.525100938	6.69E-06
GAL	1.381112341	1.198604599	1.591409961	8.00E-06
BATF	0.658512959	0.536077867	0.808911064	6.88E-05
NMU	1.371324819	1.169665985	1.607751087	9.98E-05
SLC16A1	1.424014684	1.191193307	1.702341516	0.000104137
WFS1	0.659841616	0.524863927	0.829531114	0.000370107
TFF3	0.895418014	0.842214963	0.951981923	0.00040856
TJP3	0.749823227	0.631898692	0.889754764	0.000973903
SFN	0.837556293	0.745212068	0.941343511	0.002938227
IGFBP4	0.798661755	0.679370392	0.938899615	0.006453227
HOMER2	0.778558905	0.646218792	0.938001148	0.008456115
PRKAR2B	1.360278356	1.076374498	1.719064516	0.009989895
PTPN6	0.647754779	0.458207957	0.915711407	0.013952439
NRIP1	0.74669106	0.590095794	0.944842421	0.014997896
DNAJC12	1.266086018	1.045172905	1.533692461	0.015883717
LAMC2	0.827488455	0.708750663	0.966118522	0.016569453
ANXA9	1.295296475	1.043250781	1.608235515	0.019105761
FKBP4	1.451941302	1.061142305	1.986664311	0.019757466
PPIF	1.376786995	1.050930732	1.803679701	0.020315589
CPE	1.219884181	1.031330606	1.442910165	0.020336918
GPER1	0.719663609	0.544730782	0.950773717	0.020600628
CCNA1	0.878814252	0.78644627	0.982030838	0.022607982
LARGE1	0.657064368	0.457720243	0.943225892	0.022795675
CDC20	1.289542235	1.035531979	1.605859799	0.023091816
TOP2A	1.27083213	1.029661282	1.568490851	0.025601857
ABCA3	1.234993178	1.025412355	1.487409569	0.026118785
PKP3	0.81368982	0.678303062	0.97609927	0.026384919
FGFR3	1.152817399	1.013053416	1.311863654	0.031033548
HPRT1	1.41860044	1.026698896	1.960094841	0.034032313
NPY1R	1.296879775	1.008534934	1.667663752	0.042743761
KCNK5	1.280255657	1.006308266	1.62877977	0.04430917
TRIM29	1.17795623	1.002144811	1.38461115	0.047039623
ST6GALNAC2	0.820174737	0.673517877	0.998765767	0.048580978

In this formula, the gene with a positive coefficient was a risk factor for UCEC patients, whereas the gene with a negative coefficient was a protective factor (Figure 4C).

### Evaluation of the 13 ERGs Signature as an Independent Prognostic Factor for Patients With UCEC

Next, the median risk score was used for grouping UCEC patients into low- and high-risk groups in all three groups. The Scatterplots and Heatmap were drawn to show the distribution of risk scores and the correlation between risk scores and OS, prognostic risk gene expression in the entire group. High-risk individuals were more likely to die. Some of their risk genes were overexpressed, whereas the protective genes were under-expressed (Figure 4D). The survival analysis indicated that high-risk score was significantly negatively associated with OS and DFS, whether in the Training, Validation, or Entire set (Figure 4E; Supplementary Figures S1A,B). Furthermore, whether at stage I ∼ II or stage III ∼ IV, low-risk patients all had notably favorable OS and DFS compared to high-risk ones (Supplementary Figures S1C,D). Besides, the AUCs for 1-, 3- and 5-years ROC curves in the training set were 0.73 (OS, 1 year), 0.84 (OS, 3 years), 0.82 (OS, 5 years), 0.64 (DFS, 1 year), 0.80 (DFS, 3 years), and 0.75 (DFS, 5 years), respectively (Figure 4F). Similar results were obtained in the test or Entire set (Supplementary Figures S1E,F).

Through analyzing the difference in risk scores of patient groups with different UCEC grades, stages, and TCGA subtypes, we discovered that patients with higher grades and/or stages consistently had higher risk scores (Figure 4G). Specifically, as for TCGA molecular subtypes, patients belonging to the CN-high subtype group with a relatively poor prognosis got the highest risk scores compared to the other three subtypes (Figure 4G). The Alluvial diagram showed that the high-risk group primarily corresponded to the estrogen response low ssGSEA scores group and CN-high molecular subtypes and was relevant to the poor prognosis (Figure 4H).

The above results strongly suggested that high-risk scores were significantly correlated with OS and DFS of patients with UCEC. Combined with both univariate and multivariate Cox regression analyses, the screening process identified a signature consisting of 13 DEERGs as a UCEC prognostic factor independent of clinicopathological factors such as age, grade, stage, and histological type. It proved that the risk score based on 13 DEERGs could be an independent prognostic factor for patients with UCEC, whether in the Training, Validation, or Entire set (Table 2).

**TABLE 2:** Univariate and multivariate investigation of clinic pathologic aspects for its comprehensive survival in UCEC patients.

Variables	Univariate analysis		Multivariate analysis	
	HR (95%CI)	*p* value	HR (95%CI)	*p* value
**Train set**				
Age (≥60 vs. < 60)	1.861 (1.102–3.142)	0.02	1.477 (0.848–2.572)	0.168
Histological.type (endometrial vs. mixed/serous)	2.04 (1.314–3.168)	0.001	0.867 (0.516–1.458)	0.591
Stage (III-IV vs. I-II)	1.985 (1.299–3.032)	0.002	1.854 (1.17–2.936)	0.009
Grade (G3-High_Grade vs. G1-G2)	2.173 (1.261–3.742)	0.005	1.341 (0.733–2.455)	0.341
Risk_score (High vs. Low)	3.399 (1.971–5.864)	<0.001	2.987 (1.657–5.383)	<0.001
**Test set**				
Age (≥60 vs. < 60)	1.479 (0.883–2.476)	0.137	—	—
Histological.type (endometrial vs. mixed/serous)	1.916 (1.263–2.907)	0.002	0.712 (0.443–1.147)	0.162
Stage (III-IV vs. I-II)	3.516 (2.292–5.392)	<0.001	3.321 (2.09–5.279)	<0.001
Grade (G3-High_Grade vs. G1-G2)	2.857 (1.616–5.053)	<0.001	2.095 (1.134–3.869)	0.018
Risk_score (High vs. Low)	2.982 (1.781–4.995)	<0.001	2.758 (1.602–4.747)	<0.001
**Entire set**				
Age (≥60 vs. < 60)	1.691 (1.172–2.439)	0.005	1.499 (1.021–2.201)	0.039
Histological.type (endometrial vs. mixed/serous)	2.007 (1.483–2.715)	<0.001	0.757 (0.529–1.084)	0.129
Stage (III-IV vs. I-II)	2.66 (1.975–3.582)	<0.001	2.469 (1.793–3.399)	<0.001
Grade (G3-High_Grade vs. G1-G2)	2.482 (1.676–3.676)	<0.001	1.618 (1.054–2.482)	0.028
Risk_score (High vs. Low)	3.141 (2.175–4.536)	<0.001	2.795 (1.875–4.166)	<0.001

### Establishment of the Prognostic Nomogram for the Survival of Patients With UCEC

**FIGURE 5:**
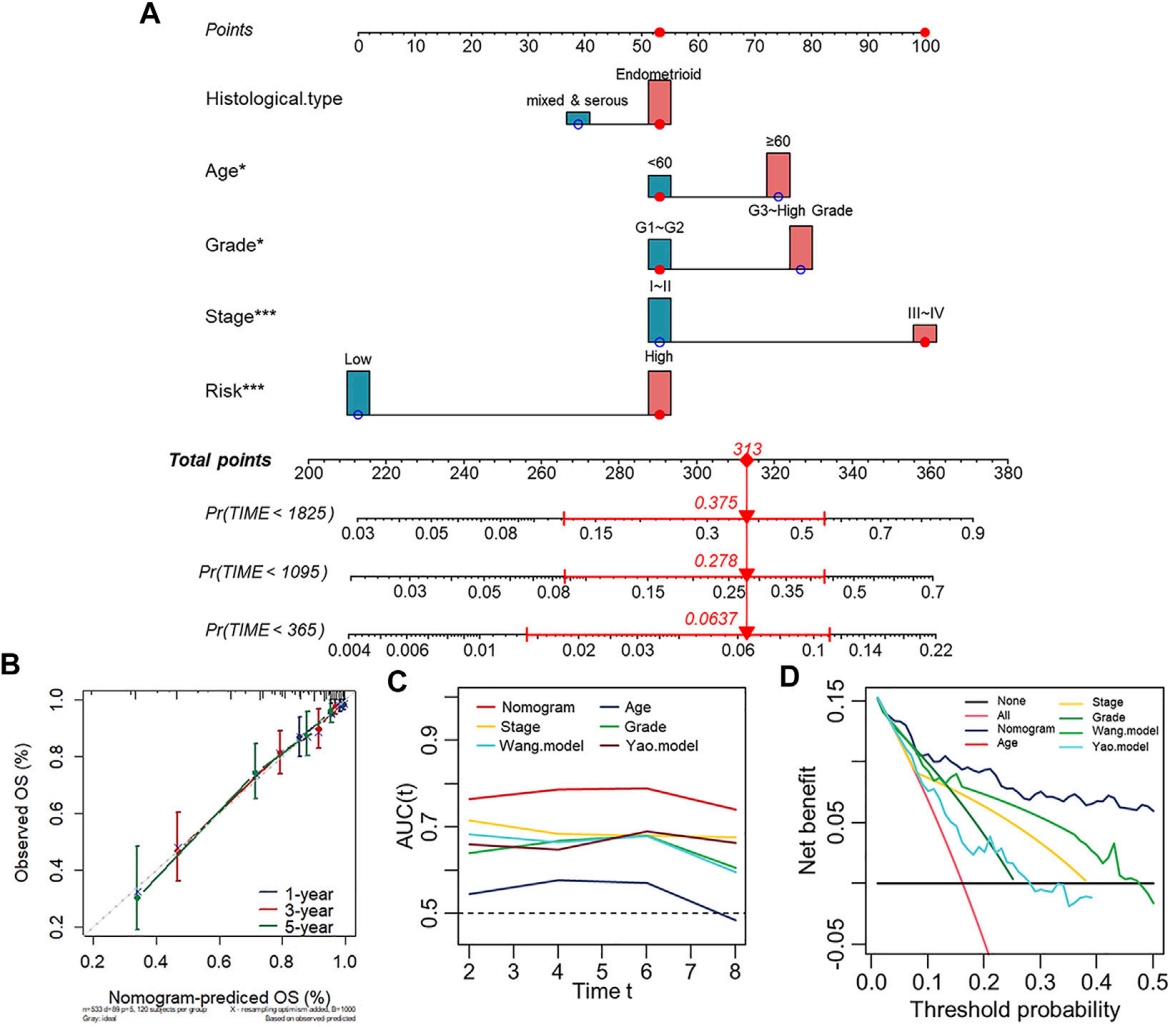
Establishment of the prognostic nomogram for the survival of patients with UCEC. **(A)** Nomogram for predicting the 1/3/5-years overall survival of patients with UCEC. **(B)** Calibration curve for the prediction of 1/3/5-years overall survival. **(C)** A comparison of time-dependent AUC curves with other clinicopathological features showing the powerful capacity for survival prediction of the nomogram. **(D)** Decision curves for 5-years OS in the Entire set.

To establish a clinically applicable model for predicting the survival probability of UCEC patients, we created a prognostic nomogram based on the risk score, age, group, and stage in the Entire set (TCGA-UCEC) to estimate the probability of the 1-, 3- and 5-years OS (Figure 5A). The calibration plots indicated the performance of the nomogram, and the 45° line represented the best predictive effect (Figure 5B). The results suggested that the nomogram performed well. Importantly, to further evaluate the predictive performance of the nomogram model in UCEC patients, we compared the nomogram model with other clinicopathological features, the Wang. model and the Yao. model (Yang et al., 2021; Chen et al., 2022). The nomogram with powerful and robust prediction performance has advantages over other clinicopathological features and models (Figure 5C). DCA graphically illustrated that the nomogram model brought more net benefit in terms of survival than other parameters and models at 5-years points (Figure 5D).

### Comprehensive Analyses of Enriched Pathways in Different Risk Groups

**FIGURE 6:**
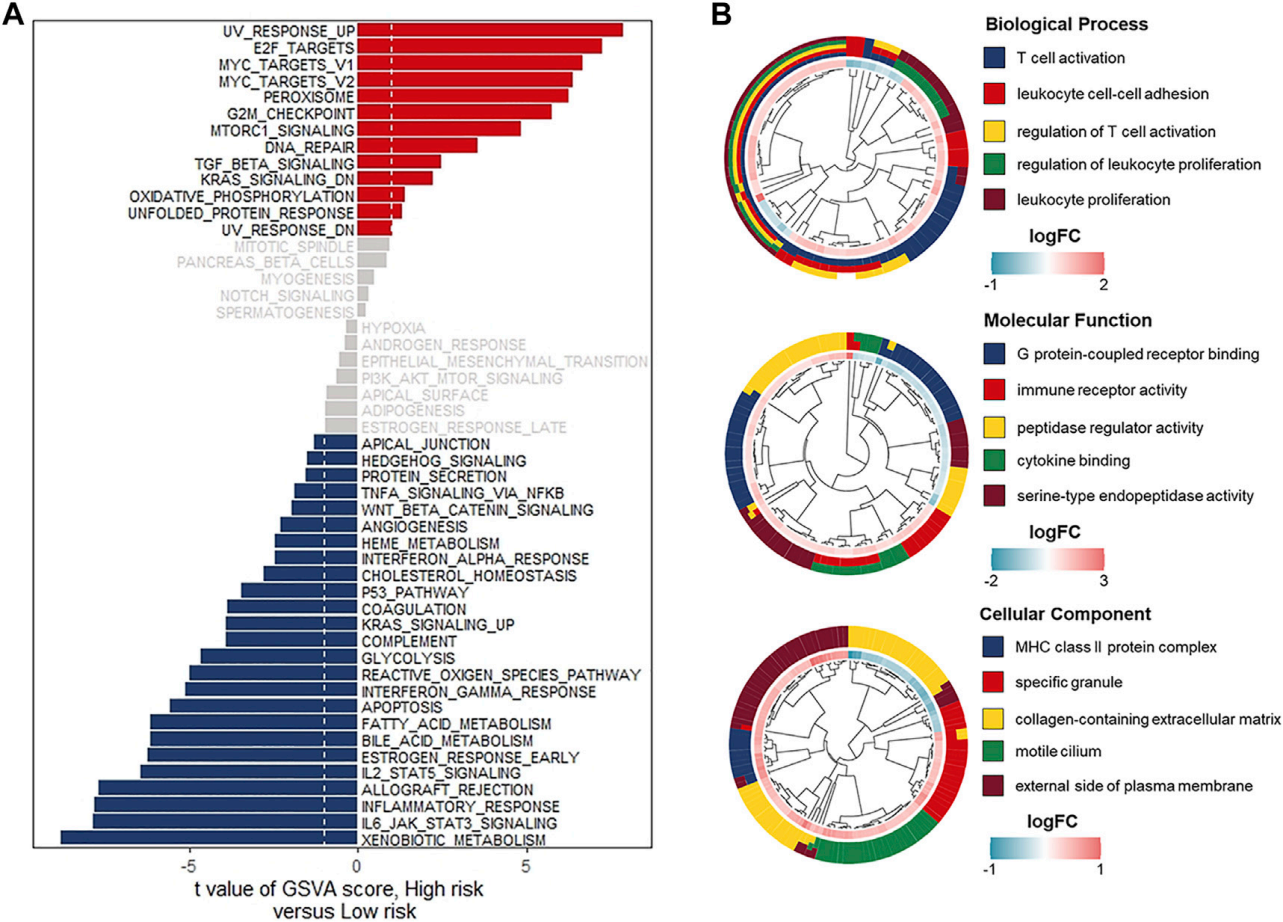
Comprehensive analyses of enriched pathways in different risk groups. **(A)** GSVA enrichment analyses in the low- and high-risk groups indicating the activated biological pathways. **(B)** GO terms analyses of 504 risk-related DEGs. Biological process (upper), Molecular function (middle), Cellular component (bottom).

To explore the differences in biological characteristics in both low- and high-risk groups, we performed GSVA enrichment analysis, which indicated that carcinogenic pathways such as G2M checkpoint, DNA repair, and TGF-beta signaling were mainly activated in the high-risk group. Whereas immune-related pathways, including the IL6-JAK-STAT3 signaling, inflammatory response, IL2-STAT5 signaling, and interferon-gamma response, were enriched in the low-risk group (Figure 6A). Comparison of transcriptional expression profiles of low- and high-risk groups identified 841 DEGs according to the screening criteria of |log2FC| > 0.5 and adj *p* -value < 0.05 (Supplementary Figure S2A). Next, the GO and KEGG pathway enrichment analyses of the 841 DEGs were performed. As the GO terms analysis showed, the top five enriched terms in biological processes were T cell activation, leukocyte cell-cell adhesion, regulation of T cell activation, regulation of leukocyte proliferation, and leukocyte proliferation. In terms of molecular function, most genes were enriched in G protein-coupled receptor binding, immune receptor activity, peptidase regulator activity, cytokine binding, and serine-type endopeptidase activity. As for the cellular component, the significantly enriched terms were MHC class II protein complex, specific granule, collagen-containing extracellular matrix, motile cilium, and the external side of the plasma membrane. (Figure 6B) Meanwhile, the KEGG pathway analysis indicated that the significantly enriched pathways were human T-cell leukemia virus 1 infection and cell adhesion molecules (Supplementary Figure S2B). Based on the above results, we suspected that relatively high immune activated status might contribute to a favorable prognosis of UCEC patients in the low-risk group.

### The Description of Immune Cell Infiltrations and the Prediction of Immunotherapeutic Response in Different Risk Groups

**FIGURE 7:**
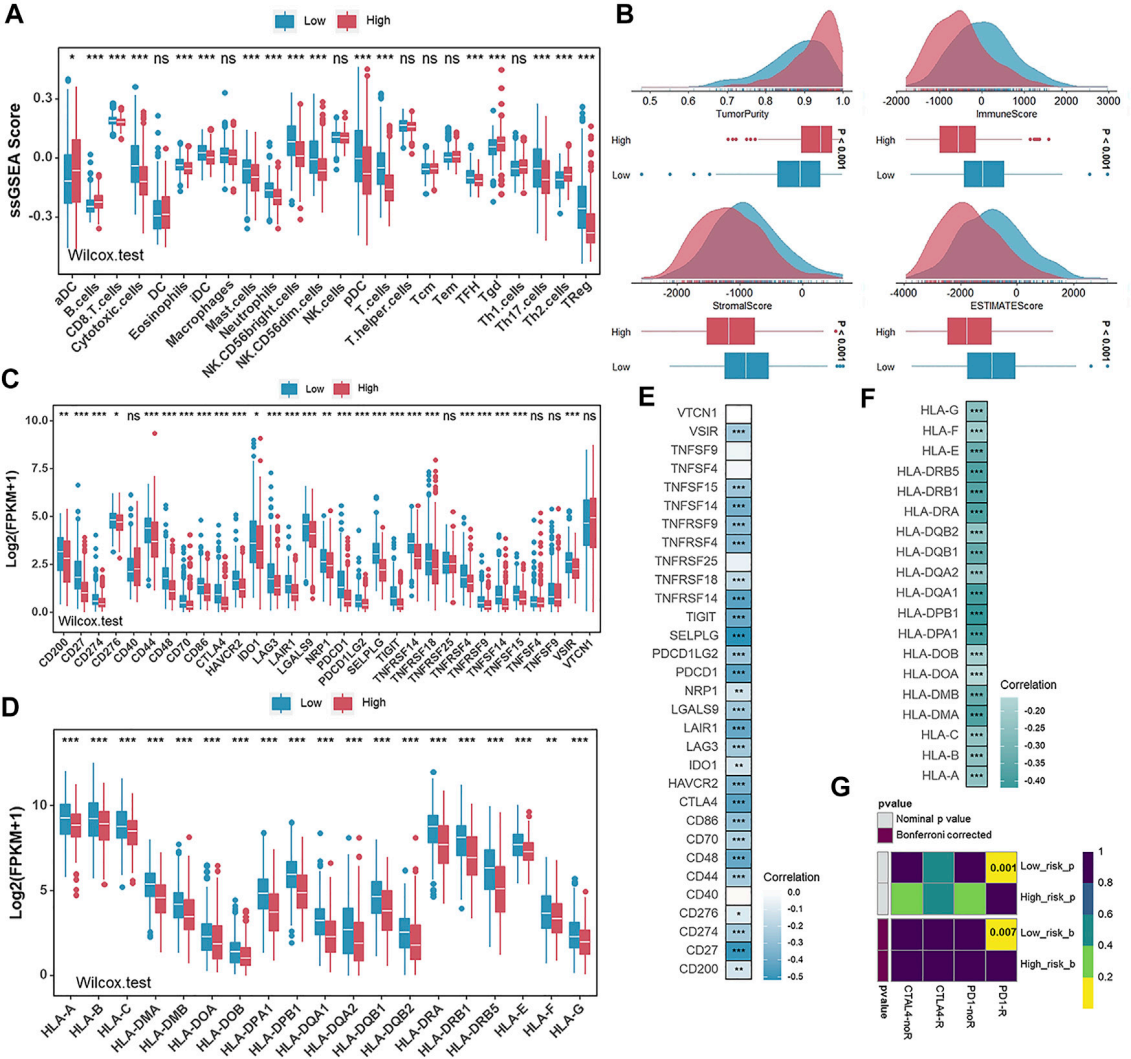
The description of infiltration of immune cells and the prediction of immunotherapeutic response in different risk groups. **(A)** The ssGSEA scores of 24 immune cells in the low- and high-risk groups in TCGA-UCEC. **(B)** Comparison of ESTIMATE score, immune score, stromal score, and tumor purity between the low- and high-risk groups in TCGA-UCEC. **(C,D)** The expression levels of immune checkpoint biomarkers**(C)** and HLA family genes **(D)** in the low- and high-risk groups in TCGA-UCEC. E and **(F)**. Correlation analysis for risk scores and the expression levels of immune checkpoint biomarkers**(E)** and HLA family genes **(F)**. **(G)** Predicted responses to anti-PD1 therapy and anti-CTLA4 therapy of patients with UCEC in the low- and high-risk groups.

Considering that the tumor microenvironment contributes to tumorigenesis and patient prognosis, we then checked the ssGSEA scores of 24 immune cell types and observed the high degree of infiltration of beneficial immune cells such as cytotoxic cells, pDC cells, T cells, and Treg cells in the low-risk group (Figure 7A). Meanwhile, the ESTIMATE algorithm was performed to calculate the Tumor Purity, ESTIMATE Score, Immune Score, and Stromal Score in the low- and high-risk groups. With relatively low Tumor Purity, the low-risk group gets a higher ESTIMATE, Immune and Stromal score when compared with the high-risk group (Figure 7B). Furthermore, we detected the expression levels of HLA family members and 31 immune checkpoint biomarkers in different risk groups. Relatively high expression levels of the above genes in the low-risk group suggested a potentially effective response to anti-immune checkpoint therapy (Figures 7C,D). The correlation between risk scores, HLA family members, and 31 immune checkpoint biomarkers was further analyzed. It was found that risk scores were significantly negatively correlated with the expression levels of HLA family members and 31 immune checkpoint biomarkers (Figures 7D,E). Therefore, we used a subclass mapping algorithm to predict the response to anti-PD1 therapy and anti-CTLA4 therapy for patients with UCEC. The Bonferroni corrected and normal *p -values* of the low-risk group were both less than 0.05, suggesting that patients from the low-risk group tended to respond effectively to anti–PD-1 therapy (Figure 7G).

### A Global Vision of Genomic Alterations and Chemotherapeutic Response Prediction in Different Risk Groups

**FIGURE 8:**
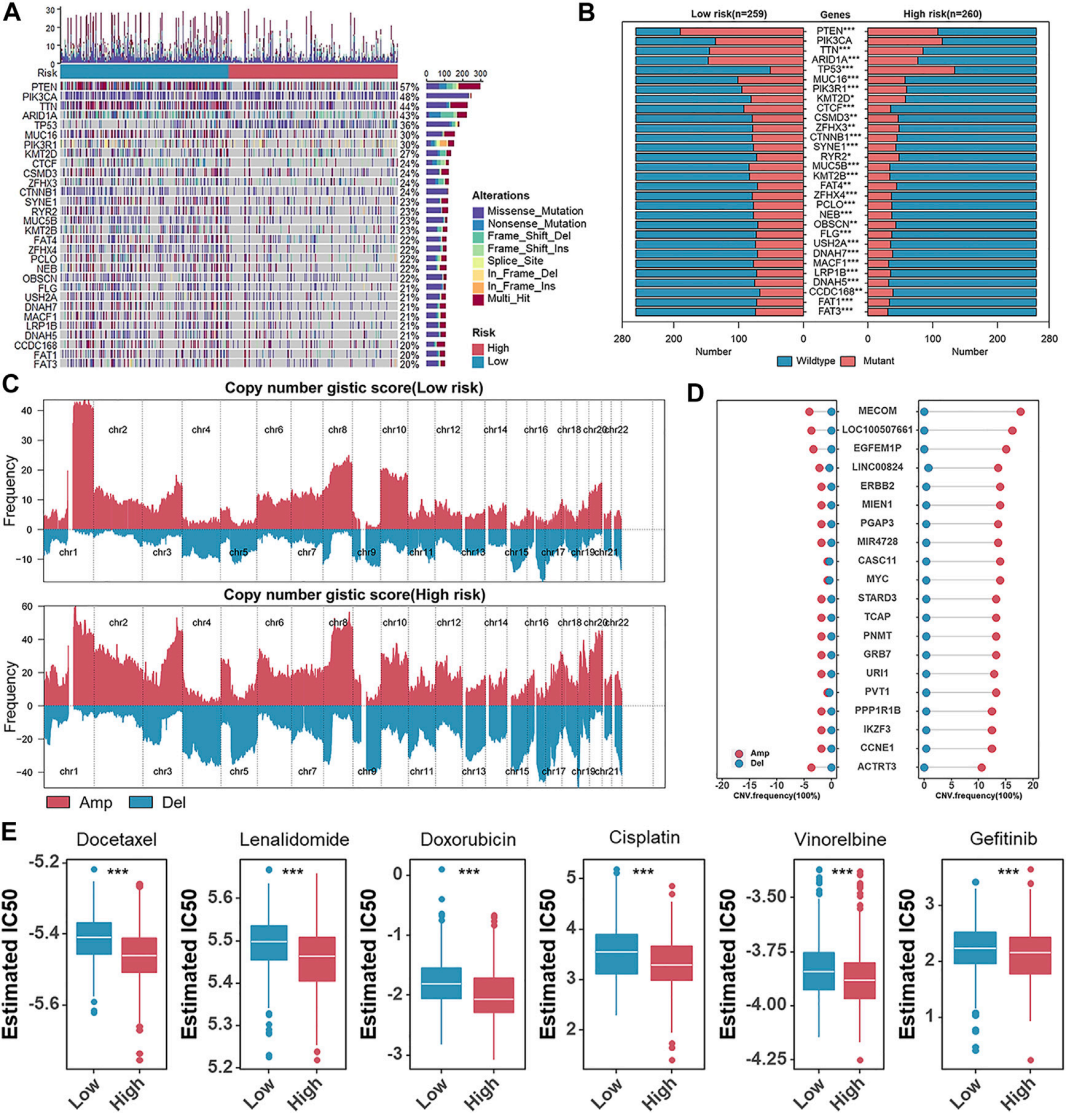
A global vision of genomic alterations and chemotherapeutic response prediction in different risk groups. **(A)** Oncoplots of the somatic mutation in TCGA-UCEC. **(B)** The box plots of top 30 somatic mutations genes in the low- and high-risk groups. **(C)**. Amplifications (Amp) and deletions (Del) of copy number in the low- and high-risk groups. **(D)** The CNV frequency of the frequently amplified (Amp) or deleted (Del) genes in TCGA-UCEC. **(E)** The box plots of the estimated IC_50_ of docetaxel, lenalidomide, doxorubicin, cisplatin, vinorelbine, and gefitinib.

There is increasing evidence that the burden of tumor mutations is related to immunotherapy in cancer patients (Samstein et al., 2019), and therefore, we detected tumor mutation burden (TMB) in different risk groups. TMB was significantly elevated in the low-risk group (Supplementary Figure S3A). To discover essential genes with somatic hypermutation that might master the oncogenesis and development of UCEC, we further analyzed the distribution of the top 30 genes in the two groups (Figure 8A). We noticed an unexpectedly higher somatic mutation frequency in the low-risk group (Figure 8B). TP53 mutations are considered a surrogate biomarker of the serous-like CN-high molecular subtype of UCEC (Singh et al., 2020). As shown in Figures 8A,B, the mutation frequency of TP53 in the high-risk group was also higher than that in the low-risk group (Figures 8A,B). In addition, the result that the CN-high subtype got the highest risk score was previously described (Figure 4E). Furthermore, we performed the GISTIC2.0 to analyze the CNV, and high-frequency amplification was discovered in the high-risk group (Figure 8C). In detail, representative amplified genes were shown, and oncogenes like MYC and CCNE1 were widely amplified in the high-risk group (Figure 8D). Considering chemotherapy as a standard treatment is currently used clinically for patients with UCEC, we used the “pRRophetic” package to estimate the patient’s sensitivity to six chemotherapeutic agents, including docetaxel, lenalidomide, doxorubicin, cisplatin, vinorelbine, and gefitinib in different risk groups. The relatively low estimated IC50 of each chemotherapeutic agent in the high-risk group indicated that although patients scored high on risk they might be more sensitive to these chemotherapeutic agents (Figure 8E).

## Discussion

Estrogens exhibit a wide range of physiological functions, including but not limited to the regulation of the menstrual cycle and reproduction to modulation of bone density, brain function, and cholesterol mobilization (Liang and Shang, 2013). The results of previous clinical, biological, and epidemiological studies have demonstrated that excessive and/or prolonged exposure to unopposed estrogen increases the risk of UCEC, especially in the endometrioid type (Zhou et al., 2019). Estrogens can signal through ERs in a genomic or nongenomic manner. Genomic signaling refers to ERs carrying out their typical steroid hormone receptor action by binding to the genome and regulating transcription. In nongenomic signaling, ERs bound to the cell surface will bind estrogens, activating other signaling pathways such as cAMP and MAPK (Losel and Wehling, 2003; Rodriguez et al., 2019). However, the mechanisms of these factors contribute to the malignant state remain unclear, despite a growing understanding of the pathophysiology and molecular biology of ERs (Zhou et al., 2019). A better understanding of ER and PR biology may make it possible to identify patient populations that are likely to benefit from new therapeutic options (Rodriguez et al., 2019).

Herein, we applied the ssGSEA algorithm to calculate the enrichment scores of gene sets of cancer-related hallmarks and found that high estrogen response ssGSEA scores indicated a better prognosis in terms of OS and DFS in UCEC patients. Furthermore, we used the LASSO Cox regression model to identify critical candidates and established a prognostic signature consisting of 13 DEERGs. Among these 13 genes, PTPN6 (Giordano et al., 2018), PGR (Zhou et al., 2020), and HPRT1 (Townsend et al., 2019) have been reported to predict the outcomes of UCEC patients. Others, including PKP3(Furukawa et al., 2005), TJP3 (Luo et al., 2020), NRIP1 (Chen X. et al., 2020), DNAJC12 (Uno et al., 2019), ASS1(Silberman et al., 2019), BATF (Feng et al., 2020), NMU (Liu et al., 2019), and IGFBP4 (Lee et al., 2018), had been verified to participate in carcinogenesis and affect patients’ prognoses in other cancers, although the relevant studies were rare in UCEC. The risk scores of UCEC patients were calculated based on the expression levels of 13 genes. Further analyses highlighted that the risk score was associated with grades, stage, and copy-number high (CN-high) subtype. Then, we divided the UCEC patients into low- and high-risk groups based on the median risk score and compared their clinicopathological parameters to clarify the correlation between the risk scores and clinical features. In the Training set, Validation set, and Entire set, the prognostic signature consisting of 13 DEERGs showed strong predictive capability and could act as a potentially independent indicator for the prognosis of UCEC patients.

To explore the biological characteristics between low- and high-risk groups, we performed GSVA enrichment analyses, which indicated that carcinogenic pathways were mainly activated in the high-risk group, whereas immune-related pathways were enriched in the low-risk group. Comparing transcriptional expression profiles of low- and high-risk groups identified 841 DEGs. Functional enrichment analyses of these DEGs could provide an understanding of their biological roles. In the GO analysis, both immune cell adhesion and activation and antigen presentation and binding were associated with the risk-related DEGs. Mounting evidence has shown that ERs are broadly expressed in many cell types involved in innate and adaptive immune responses (Rothenberger et al., 2018). Based on the above analyses, we hypothesized that a prognostic signature consisting of 13 DEERGs might be associated with immune cell infiltration and help guide therapeutic regimens.

In order to verify our hypothesis, the ssGSEA algorithm was used to evaluate the immune cell fraction, and ESTIMATE was used to evaluate Tumor Purity, ESTIMATE Score, Immune Score, and Stromal Score in UCEC patients. Many elegant studies have revealed that effectors, including CTLs, B cells, and NK cells, destroy tumor cells while myeloid-derived suppressor cells (MDSCs) and tumor-associated macrophages (TAMs) can contribute to immune escape and tumor growth (Taube et al., 2018). The low-risk group had relatively lower Tumor Purity and higher ESTIMATE, Immune, and Stromal Scores. Moreover, this group of patients was remarkably rich in cytotoxic cells, pDC cells, T cells, and Treg cells. Regulatory T cells (Tregs) are potent immunosuppressive lymphocytes that are crucial for immune tolerance and homeostasis (Adair et al., 2017). However, the function of Tregs in endometrial cancer is still controversial (Prieto, 2011). Bingnan Chen et al. found that the infiltrated level of Tregs was positively correlated with the survival rate of endometrioid endometrial adenocarcinoma and negatively correlated with clinical grading (Chen B. et al., 2020). Our study also found that the infiltrated Tregs level was elevated in the low-risk group with a better prognosis. The reason is still unclear and may be associated with the level of estrogen (Chen B. et al., 2020). T cells can recognize neoantigens *via* HLA molecules on the tumor cell surface, which provides an opportunity to initiate specific and effective anti-cancer immune responses (Liu et al., 2020). The results showed that HLA genes were significantly higher in the low-risk group. In addition, we observed that the low-risk group was significantly associated with elevated immune checkpoint levels, implying the potential predictive value of immunotherapy benefits. Based on the background mentioned before, we used a subclass mapping algorithm to predict the response of UCEC patients to immunotherapy. We found that patients in the low-risk group were more likely to respond effectively to anti-PD-1 immunotherapy. Previous studies demonstrated that the activation of the estrogen pathway could enhance macrophage influx, Th2 responses, and immunosuppression by NK and CTL-mediated tumor cell elimination (Jiang et al., 2006; Jiang et al., 2007; Khan and Ansar Ahmed, 2015; Svensson et al., 2015). Therefore, anti-estrogen therapy combined with immunotherapy should be considered an effective therapeutic regimen for patients with UCEC.

An assessment of the mutated genes underlying human tumors is essential to cancer diagnosis, therapy, and rational treatment selection (Chong et al., 2021). Tumors containing p53 mutations exhibit a high degree of genomic instability associated with tumor progression and invasion by upregulation of p53-mutant target genes, and the TP53 mutation is well known for its prognostic impact in endometrial carcinoma (Stelloo et al., 2016). Previous studies demonstrated that the CN-high molecular subtype of endometrial carcinoma was characterized by the TP53 mutation and frequently accompanied by many gene copy-number alterations, including the amplifying of essential oncogenes such as CCNE1 and c-MYC (Leskela et al., 2019). In our present study, the high-risk group primarily corresponded to the CN-high molecular subtype and was significantly correlated with more aggressive molecular changes such as frequent TP53 mutations and extensive copy number alterations. High TMB indicates that cancer cells have a high level of mutations, suggesting that cancer cells are more different from normal cells, which can be easily discovered by the human immune system (Zhao et al., 2021). In our study, the low-risk groups displayed more mutation frequency and a higher degree of infiltration of immune cells.

Despite some exciting discoveries, some problems remain. First, this study is a retrospective study only covering the TCGA dataset and thus should be validated by an external dataset and further confirmed by preliminary experiments. Second, the predictive capacity of the prognostic signature composed of 13 genes and its potential relationship with immune status, requires further verification testing in other clinical samples.

## Conclusion

In this study, we developed an estrogen-response-related signature that could act as an independent prognostic factor for patients with UCEC. According to the prognostic model based on 13 DEERGs, we comprehensively evaluated the biological behaviors, immune status, genomic alterations, and therapeutic responses in different risk groups. In summary, our study provides a novel insight into potential strategies for diagnosing, monitoring, and treatment of UCEC.

## Data Availability

The original contributions presented in the study are included in the article/Supplementary Material, further inquiries can be directed to the corresponding authors.

## Abbreviations

CNV, copy number variation; DEERGs, differentially expressed estrogen-response-related genes; LASSO, least absolute shrinkage and selection operator; PCA, principal component analysis; ssGSEA, single-sample gene set enrichment analysis; TCGA, the cancer genome atlas; TMB, tumor mutational burden; UCEC, uterine corpus endometrial carcinoma.
