# Supplementary material for: A 13-Gene Signature Based on Estrogen Response Pathway for Predicting Survival and Immune Responses of Patients With UCEC
Source: Front Mol Biosci. 2022 Apr 26;9:833910. doi: 10.3389/fmolb.2022.833910 (PMC9087353; doi:10.3389/fmolb.2022.833910)
Supplement: Supplementary file 1 [file DataSheet1.pdf]

SUPPLEMENTAL FIGURES

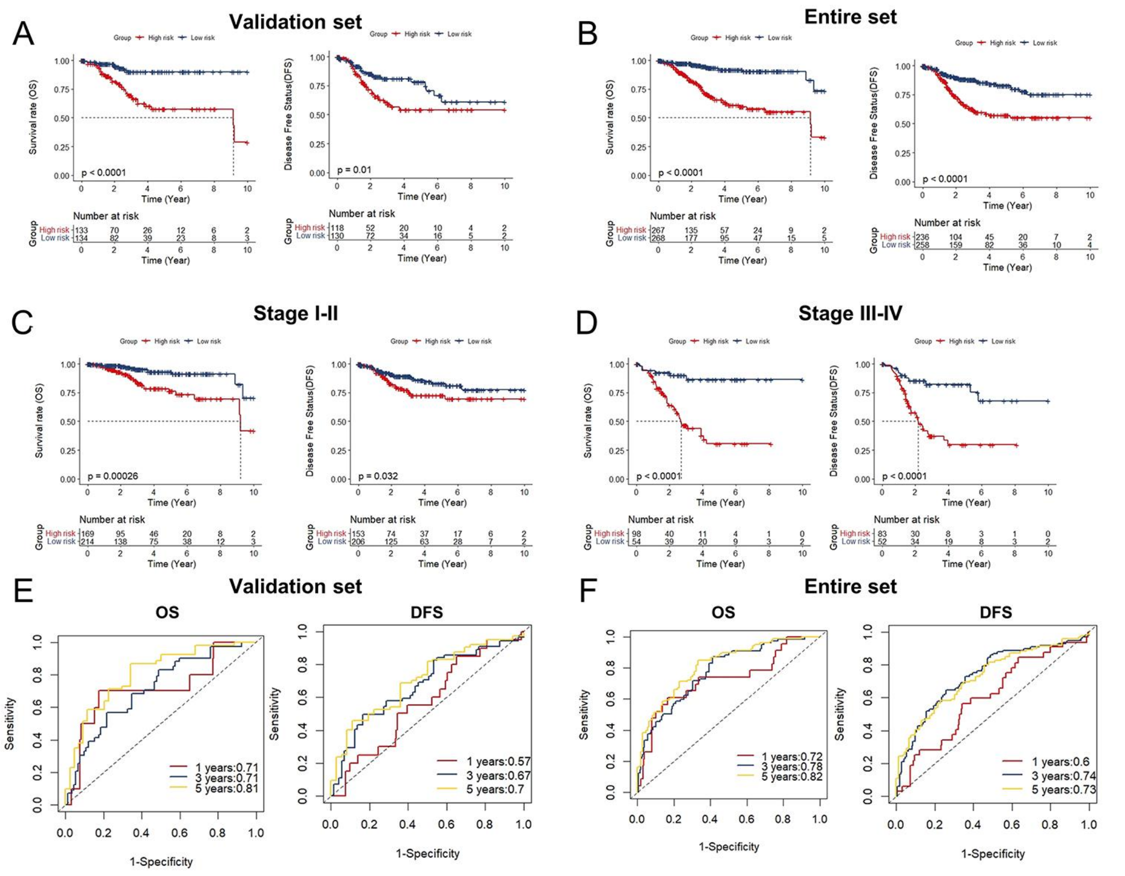

Supplement 1. Survival analyses of the low- and high-risk groups patients.

A, B. Kaplan–Meier survival analyses of the low- and high-risk groups patients in the validation set (A) and the entire set (B).

C, D. Kaplan–Meier survival analyses of the low- and high-risk groups patients at stage I~II (C) and stage III~IV (D).

E, F. ROC curve analyses according to the 1, 3, 5-year survival of the area under the AUC value in the validation set(E) and the entire set(F).

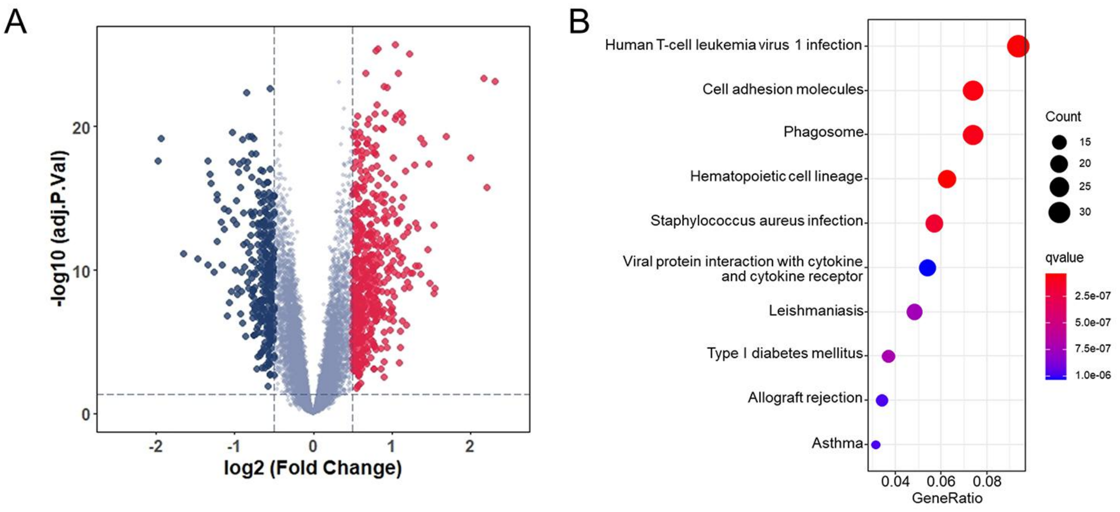

Supplement 2. Comprehensive analyses of enriched pathways in different risk groups.

A. Volcano plot representing 504 risk-related DEGs between low- and high-risk groups.

B. KEGG analysis of 504 risk-related DEGs. The size of the balls indicating the number of genes and color indicating q value.

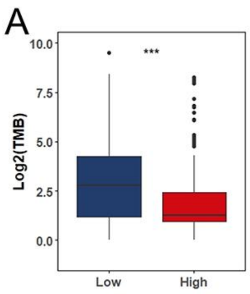

Supplement 3. Comparison of genomic alterations in different risk groups

A. The tumor mutation burden in the low- and high-risk groups.
